# Life Time Saga with Custom Mega Prosthesis in Bone Tumors (The Chennai Experience)

**DOI:** 10.1007/s13193-024-01915-z

**Published:** 2024-04-12

**Authors:** Mayilvahanan Natarajan

**Affiliations:** 1 Cancer Institute (WIA), Adyar, Chennai, Tamil Nadu India; 2 Dept. of Orthopaedic Surgory, Madras Medical College & Govt. Gen. Hospital, P H Road, Park Town, Chennai, Tamilnadu 600003 India

In the landscape of cancer treatment, the journey towards effective management and improved patient outcomes has been fraught with challenges. Prior to the pioneering efforts of individuals like Dr. Mayilvahanan Natarajan, the field of Orthopaedic Oncology in India faced daunting obstacles, marked by limited access to advanced treatments and prohibitive costs of specialized implants. Patients grappling with bone sarcomas encountered the grim reality of limb amputations as the primary mode of surgical intervention, with few options for limb salvage surgeries. This article delves into the transformative impact of Dr. Natarajan’s innovations and collaborations, which not only revolutionized bone sarcoma treatment but also offered a ray of hope to countless patients across the country.

Dr. Mayilvahanan Natarajan pursued his graduate and post-graduate education in Orthopaedic Surgery at the Premier Institute in Tamil Nadu, the Madras Medical College and Govt. General Hospital (1971–1981). He trained in the prestigious Royal Liverpool Hospital, UK in 1985. He joined the Cancer Institute (WIA) as a Visiting Consultant in 1988 and served the Institute until 2015 (27 years).

Surgical management of malignant bone tumours like Osteosarcoma and Ewing sarcoma primarily was by ablation of the Limb. Limited Limb Salvage procedures were performed, notably by Prof. M. Natarajan (1957–1976) wherein aggressive giant cell tumour of distal radius was resected and replaced by a fibular graft. Prof. T. K. Shanmugasundaram (1976–1987) employed techniques such as the turn-up turn-down graft for aggressive GCT around the knee. Low-grade malignant tumours like chondrosarcoma were resected and stabilised with fibular grafts.

Limb Salvage surgeries were more advanced in the UK, where Dr. Mayilvahanan received training at Swindon and Liverpool (1984–1985). True advancement in Orthopaedic Oncology began in 1988 when Dr. Mayilvahanan requested Dr. Krishnamurthy and Dr. Shantha of the Adyar Cancer Institute (WIA) to permit him to establish Orthopaedic Oncology Services at the Cancer Institute to which they readily agreed.

The focus was on treating malignant tumours by wide resection and replacement with Custom Mega Prosthesis. The high cost of the imported Custom-Made Prostheses from Howmedica (INR 3,00,000) made it imperative that a cost-effective Indian version (INR 75,000) be made out of medical-grade stainless steel alloy.

Further inspiration came from attending the International Society of Limb Salvage Surgery (ISOLS) Meeting, particularly the 5^th^ meeting in San Malo, France, in 1989. The exposure to cutting-edge advancements in reconstruction techniques, both biological and prosthetic, motivated Dr. Mayilvahanan to continue to design and develop the indigenous Custom Mega Prostheses.

The milestone event occurred on Vijayadasami day, 20^th^ October 1988, when the first patient underwent distal femur resection and replacement with a Custom-Made Mega Prosthesis for an aggressive GCT in the distal femur. This marked a significant moment in the history of Musculo Skeletal Oncology in India, as it demonstrated the feasibility of such procedures. Subsequently, this led to an influx of patients from all over India seeking Limb Salvage Surgery by Custom Prosthesis.

Addressing the challenge of prosthetic durability, Mr. Jayasingh and Mr. Micheal collaborated to design robust stainless steel custom implants. This partnership led to the establishment of Arc Bio Mechanical Engineers, which provided affordable implants, thereby solidifying Cancer Institute and Govt. General Hospital Chennai as a leading centre for bone sarcoma treatment by Custom Mega Prosthesis in the country.

The locally made Custom Mega Prosthesis was available only in Chennai. The Cancer Institute (WIA) and Govt. General Hospital were the only centres doing this specialised work between the years 1988 to 2000. A series of 400 patients of malignant bone tumours were treated during this period by Custom Mega Prosthetic replacement by Dr. Mayilvahanan Natarajan. After the year 2000, centres in Mumbai, Delhi and Calcutta started doing the custom prosthetic replacement for their tumour patients in India.

This pioneering work with locally made and cost-effective implants was recognized internationally at the 3^rd^ meeting of the Asia Pacific Musculo-Skeletal Tumour Society in Hong Kong in the year 2000. Dr. Mayilvahanan Natarajan was made President of the APMSTS, leading to India hosting the 4^th^ Annual Conference of the APMSTS in Chennai in the year 2002. This was a significant milestone in the development of Orthopaedic Oncology in India.

The 10-years follow-up results of the longevity of the indigenous Custom Prosthesis proved that the prosthesis was able to withstand the test of time and was comparable to the imported prosthesis [1–5]. Advancements in chemotherapy, including intra-arterial chemotherapy and standardized regimens, significantly improved patient outcomes, particularly in Ewing’s sarcoma.

Continuing his work at the Cancer Institute (WIA) and the Department of Orthopaedics, Govt. General Hospital, his series of Limb Salvage with Custom Mega Prosthesis grew up to 2292 in 36 years [6–10]. Travelling the length and breadth of India, attending the National Orthopaedic Conferences and presenting his work, he propagated the technique and technology of Custom Mega Prosthesis to several states in India. His publications in peer-reviewed International Journals showed the long-term results of the Indigenous Custom Mega prosthesis were comparable with those from other countries.

Transitioning from stainless steel alloy to titanium alloy prostheses enhanced durability and reduced fracture risks. This transformation was facilitated by our former President of India respectful Dr. APJ Abdul Kalam. The Mishra Dhatu Nigam Limited (MIDHANI) started to manufacture and supply titanium implants nationwide.

Biological grafts gained importance, leading to the establishment of the First Bone Bank in the State at the Government General Hospital in the year 2005, followed by a second one at the Cancer Institute (WIA) in 2019. The use of liquid nitrogen for sterilization and re-implantation of tumour-bearing bone autografts was pioneered at the Cancer Institute (WIA), alongside innovative techniques like pedicled cryotherapy for autograft reconstruction.

Dr. Mayilvahanan Natarajan’s tireless efforts and groundbreaking contributions to Orthopaedic Oncology were recognized with the Dr. B.C. Roy National Award (2004), National Award for Welfare of the Disabled (2005) and the Padma Shri Award (2007) by the President of India.

Through pioneering collaborations between the Cancer Institute (WIA) and the Government General Hospital, Chennai, advanced treatments were made more accessible across the country [11–15]. The tireless advocacy and propagation of these techniques have not only democratized access to orthopaedic oncology care but also positioned India as a leading centre for bone sarcoma treatment globally.

In conclusion, the journey of Orthopaedic Oncology in India, spearheaded by Dr. Mayilvahanan Natarajan, reflects a remarkable evolution marked by innovation, collaboration and a relentless pursuit of improving patient care and outcomes. By self-design and indigenous manufacturing Dr. Mayilvahanan Natarajan has made the Custom Mega Prosthesis Affordable to patients, acceptable to the medical fraternity, applicable to society and the concept of custom mega prosthesis in bone tumour attainable in our country, so that India keeps pace with the rest of the world in Orthopaedic Oncology [16–18]. Recognized with prestigious awards, his legacy serves as a beacon of hope for the future of Orthopaedic Oncology, inspiring continued innovation and dedication to improving patient outcomes, ensuring that India remains at the forefront of this critical medical field.
